# Maintaining the unmethylated state

**DOI:** 10.1186/1868-7083-5-17

**Published:** 2013-09-30

**Authors:** Steven S Smith

**Affiliations:** 1 City of Hope, 1500 East Duarte Road, Duarte, CA 91010, USA

## Abstract

**Background:**

A remarkable correspondence exists between the cytogenetic locations of the known fragile sites and frequently reported sites of hypermethylation. The best-known features of fragile sites are sequence motifs that are prone to the spontaneous formation of a non-B DNA structure. These facts, coupled with the known enzymological specificities of DNA methyltransferase 1 (DNMT1), the ATP-dependent and actin-dependent helicases, and the ten-eleven translocation (TET) dioxygenases, suggest that these enzymes are involved in an epigenetic cycle that maintains the unmethylated state at these sites by resolving non-B structure, preventing both the sequestration of DNA methyltransferases (DNMTs) and hypermethylation in normal cells.

**Presentation of the hypothesis:**

The innate tendency of DNA sequences present at fragile sites to form non-B DNA structures results in *de novo* methylation of DNA at these sites that is held in check in normal cells by the action of ATP-dependent and actin-dependent helicases coupled with the action of TET dioxygenases. This constitutes a previously unrecognized epigenetic repair cycle in which spontaneously forming non-B DNA structures formed at fragile sites are methylated by DNMTs as they are removed by the action of ATP-dependent and actin-dependent helicases, with the resulting nascent methylation rendered non-transmissible by TET dioxygenases.

**Testing the hypothesis:**

A strong prediction of the hypothesis is that knockdown of ATP-dependent and actin-dependent helicases will result in enhanced bisulfite sensitivity and hypermethylation at non-B structures in multiple fragile sites coupled with global hypomethylation.

**Implications of the hypothesis:**

A key implication of the hypothesis is that helicases, like the lymphoid-specific helicase and alpha thalassemia/mental retardation syndrome X-linked helicase, passively promote accurate maintenance of DNA methylation by preventing the sequestration of DNMTs at sites of unrepaired non-B DNA structure. When helicase action is blocked due to mutation or downregulation of the respective genes, DNMTs stall at unrepaired non-B structures in fragile sites after methylating them and are unable to methylate other sites in the genome, resulting in hypermethylation at non-B DNA-forming sites, along with hypomethylation elsewhere.

## Background

Our recent work on the mechanism of action of 2’-deoxyriboguanylurea (GuaUre-dR) [1], the primary breakdown product of 5-aza-2’-deoxycytidine (5azaC-dR) [2], coupled with work from multiple laboratories, as well as our own, on DNA methyltransferases (DNMTs) [3-8], the substrate specificity, mechanism of action and biological effects of helicases, such as the ERCC2, ATRX, HELLS and RecQ family of helicases [9-15], and the ten-eleven translocation (TET) dioxygenases [16-19], suggest that the mechanism responsible for most of the hypermethylation observed during carcinogenesis involves the breakdown of an epigenetic repair cycle that maintains the unmethylated state at and near the common fragile sites.

**Figure 1:**
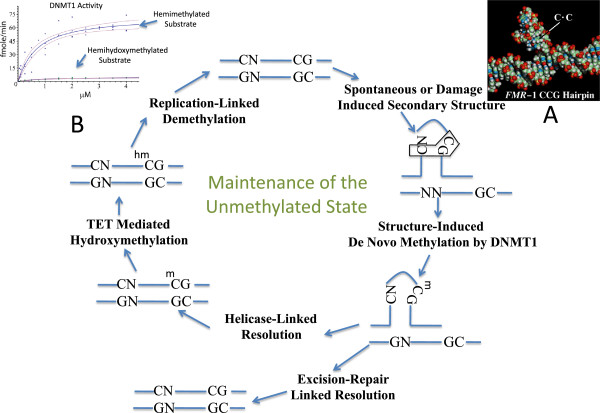
**Key systems maintaining the active and unmethylated state of DNA at sequences with the innate potential for non-B DNA structure formation.** In this enzymologically-based model, non-B DNA structure forms spontaneously or in response to replication stress or carcinogen-linked damage, inducing DNA methylation *de novo*[3,4,103,104]*.* The three-nucleotide recognition motif [4] of DNMT1 (C:G-C) is highlighted in the schematic of the non-B structure in the upper right of the figure. Helicase resolution at non-B structures produces hemimethylated DNA. Hypermethylation is prevented by the action of TET dioxygenase on its preferred hemimethylated substrate [17]. When stress overwhelms the capacity of TET dioxygenase to hydroxymethylate hemimethylated DNA in the affected region, hypermethylation will result. In this model, helicase lesions, DNMT lesions or TET dioxygenase lesions are expected to generate chromosome instability and the selective induction of fragile sites. Methylated unusual DNA structures that are not resolved by helicase action may be removed by excision repair-linked pathways where the unmethylated state is restored by DNA synthesis. **(A)** Molecular model of the hypermethylated C-rich strand hairpin formed at fragile site FRAXA. The model was constructed in Biograf 3.1 (Molecular Simulations Inc, San Diego, CA, USA) and rendered with the UCSF Chimera package (Resource for Biocomputing, Visualization, and Informatics, University of California, San Francisco, CA, USA). It is based on NMR data presented by Chen *et al.*[20]. **(B)** Activity of human DNA methyltransferase 1 (hDNMT1) on hemimethylated DNA and hemihydroxymethylated DNA. hDNMT1 was purified from nuclear extracts [105] prepared from cultured HeLa S3 cells. Purification and enzyme activity measurements were carried out as previously described [106,107]. The purified enzyme had a specific activity of 20.96 fmole^3^HCH_3_/min/mg. Duplex ODN substrates were synthesized and annealed as previously described [107]. The results confirm the findings of Hashimoto *et al*. [17] with cloned DNMT1.

The classic examples of epigenetic downregulation in human cells and tissues are genes that are often silenced and hypermethylated during tumorigenesis. As demonstrated in Table 1, the vast majority of these genes reside at cytogenetic locations that define well-known fragile sites. This remarkable cytogenetic correspondence strongly suggests that hypermethylation, epigenetic downregulation and chromosomal fragility share common mechanistic features. The best-known feature of fragile sites is the presence of a sequence motif that is prone to the spontaneous formation of a non-B DNA structure. In addition to FRAXA [14], many other fragile sites have been shown to harbor sequences, such as the CCG triplet repeat, which form hairpins, slippage intermediates (Figure 1A) and quadruplex structures. Non-B intermediates are known to be exceptional substrates for *de novo* methylation by DNA methyltransferase 1 (DNMT1) [6,7,20] either at its three-nucleotide recognition motif (Figure 1) within the repeat if it contains CG sites or at the same motif at CG sites flanking the non-B sequence if it does not. Consequently, even fragile sites that contain AT-rich sequences with high torsional flexibility and the potential for non-B DNA structure formation are subject to methylation in regions flanking the repeat. Other fragile sites that lack CG dimers, such as the Huntington’s disease CAG repeat, which can also form hairpins and slippage intermediates [7,21], appear to induce methylation at the flanking and other regions where CG dimers occur [7,22]; for a review, see Lukusa and Fryns [23].

**Table 1:** Hypermethylation at known fragile sites

**Gene**	**Gene location**	**Fragile site**	**Fragile site type**	**Fragile site location**	**Methylation reference**
*RUNX3*	1p36	FRA1A	Aph, C	1p36	[24]
-	-	FRA1B	Aph, C	1p32	-
*ARHI*	1p31	FRA1C	Aph, C	1p31.2	[25]
*PARG1*	1p22.1	FRA1D	Aph, C	1p22	[26]
-	-	FRA1E	Aph, C	1p21.2	-
*S100A6*	1q21	FRA1F	Aph, C	1q21	[27]
*-*	-	FRA1G	Aph, C	1q25.1	-
*PTGS2*	1q25.2-25.3	FRA1G	Aph, C	1q25.1	[28,29]
*DISC1*	1q42.1	FRA1H	5azaC-R, C	1q42	[30]
-	-	FRA1I	Aph, C	1q44	-
-	-	FRA1J	5azaC-R, C	1q12	-
*GLUL*	1q31	FRA1K	Aph, C	1q31	[31]
*CLCA2*	1p31	FRA1L	Aph, C	1p31	[32]
*MIR137*	1p21.3	FRA1M	Fol, R	1p21.3	[33]
-	-	FRA2A	Fol, R	2q11.2	-
*BCL2L11*	2q13	FRA2B	Fol, R	2q13	[34]
-	-	FRA2C	Aph, C	2p24.2	-
*MSH6*	2p16	FRA2D	Aph, C	2p16.2	[35]
*DOK1*	2p13	FRA2E	Aph, C	2p13	[36]
*RASSF1*	3p21.3	-	-	-	[37]
*MLH1*	3p21.3	-	-	-	[38]
*VHL*	3p25.3	-	-	-	[39]
*LRP1B*	2q21.2	FRA2F	Aph, C	2q21.3	[40]
*HOXD1*	2q31.1	FRA2G	Aph, C	2q31	[41]
-	-	FRA2H	Aph, C	2q32.1	-
*FLIP*	2q33-q34	FRA2I	Aph, C	2q33	[42]
*KIF1A*	2q37.3	FRA2J	Aph, C	2q37.3	[43]
*ZEB2*	2q22.3	FRA2K	Fol, R	2q22.3	[44]
*RARβ*	3p24.2	FRA3A	Aph, C	3p24.2	[45]
*FHIT*	3p14.2	FRA3B	Aph, C	3p14.2	[46]
-	-	FRA3C	Aph, C	3q27	-
*RARRES1*	3q25.32	FRA3D	Aph, C	3q25	[47]
*PTX3*	3q25	FRA3D	Aph, C	3q25	[48]
-	-	FRA4A	Aph, C	4p16.1	-
*PDGFRA*	4q12	FRA4B	BrdU, C	4q12	[49]
*SFRP2*	4q31.3	FRA4C	Aph, C	4q31.1	[50]
*SLIT2*	4p15.2	FRA4D	Aph, C	4p15	[51]
-	-	FRA5A	BrdU, C	5p13	-
-	-	FRA5B	BrdU, C	5q15	-
*NR3C1*	5q31.3	FRA5C	Aph, C	5q31.1	[52]
*GPR150*	5q15	FRA5D	Aph, C	5q15	[53]
-	-	FRA5E	Aph, C	5p14	-
*APC*	5q21-22	FRA5F	Aph, C	5q21	[54]
-	-	FRA5G	Fol, R	5q35	-
*SCGB3A1*	5q35.3	FRA5G	Fol, R	5q35	[55,56]
-	-	FRA6A	Fol, R	6p23	-
-	-	FRA6B	Aph, C	6p25.1	-
-	-	FRA6C	Aph, C	6p22.2	-
-	-	FRA6C	Aph, C	6p22.2	-
-	-	FRA6D	BrdU, C	6q13	-
*ESR1*	6q25.1	-	-	-	[57]
-		FRA6E	Aph, C	6q26	-
*HACE1*	6q21	FRA6F	Aph, C	6q21	[58]
-	-	FRA6G	Aph, C	6q15	-
-	-	FRA7A	Fol, R	7p11.2	-
*TWIST1*	7p21.2	-	-	-	[56]
-	-	FRA7B	Aph, C	7p22	
-	-	FRA7C	Aph, C	7p14.2	-
*IGFBP3*	7p13-p12	FRA7D	Aph, C	7p13	[59]
*HIC1*	17p13.3	FRA7D	Aph, C	7p13	[60]
*ABCB1*	7q21.12	FRA7E	Aph, C	7q21.2	[61]
*TFPI2*	7q22	FRA7F	Aph, C	7q22	[62]
*TES*	7q31.2	FRA7G	Aph, C	7q31.2	[63]
*CFTR*	7q31.2	FRA7G	Aph, C	7q31.2	[64]
-	-	FRA7H	Aph, C	7q32.3	-
*EN2*	7q36	FRA7I	Aph, C	7q36	[65]
*HSPB1*	7q11.23	FRA7J	Aph, C	7q11	[66]
-	-	FRA8A	Fol, R	8q22.3	-
-	-	FRA8B	Aph, C	8q22.1	-
*MYC*	8q24.21	FRA8C,	Aph, C	8q24.1	[67]
-	-	FRA8D	Aph, C	8q24.3	-
*MYC*	8q24.21	FRA8E	Dmy, R	8q24.1	[67]
*CDKN2A*	9p21	FRA9A,	Fol, R	9p21	[68]
*CDKN2B*	9p21	FRA9A,	Fol, R	9p21	[69]
*BRINP1*	9q32-q33	FRA9B,	Fol, R	9q32	[70]
*CDKN2A*	9p21	FRA9C,	BrdU, R	9p21	[68]
*CDKN2B*	9p21	FRA9C,	BrdU, R	9p21	[69]
*DAPK1*	9q21.33	FRA9D	Aph, C	9q22.1	[71]
		FRA9B,	Aph, C	9q32	
*BARX1*	9q12	FRA9F	5azaC-R, C	9q12	[72]
*FRA10AC1*	10q23.33	FRA10A	Fol, R	10q23.3	[73]
*PTEN*	10q23.3	FRA10A	Fol, R	10q23.3	[74]
-		FRA10B	BrdU, R	10q25.2	
		FRA10C	BrdU, R	10q21	
*EGR2*	10q21.1	FRA10D	Aph, C	10q21.1	[75]
*TYSND1*	10q22.1	FRA10D	Aph, C	10q22.1	[76]
-	-	FRA10E	Aph, C	10q25.2	
*MGMT*	10q26	FRA10F	Aph, C	10q26.1	[77]
*RET*	10q11.2	FRA10G	Aph, C	10q11.2	[78]
*MRPL48*	11q13.4	FRA11A	Fol, R	11q13.3	[79]
*AMICA1*	11q23.3	FRA11B	Fol, R	11q23.3	[80]
*CALCB*	11p15.2-	FRA11C	Aph, C	11p15.1	[81]
*HRAS*	11p15.5	FRA11C,	Aph, C	11p15.1	[81]
*MYOD1*	11p15.4	FRA11C,	Aph, C	11p15.1	[64]
-	-	FRA11D	Aph, C	11p14.2	
*WT1*	11p13	FRA11E	Aph, C	11p13	[82]
*CD44*	11p13	FRA11E	Aph, C	11p13	[83]
-	-	FRA11F	Aph, C	11p14.2	
*PGR*	11q22-q23	FRA11G	Aph, C	11q23.3	[84]
*GSTP1*	11q13	FRA11H	Aph, C	11q13	[85]
*CCND2*	12p13	-	-	-	[56]
*CALCB*	11p15.2-15.1	FRA11I	Aph, C	11p15.1	[81]
*HRAS*	11p15.5	FRA11I,	Aph, C	11p15.1	[81]
*MYOD1*	11p15.4	FRA11I,	Aph, C	11p15.1	[64]
-	-	FRA12A	Fol, R	12q13.1	
*SLC6A15*	12q21.3	FRA12B	Aph, C	12q21.3	[62]
*CHFR*	12q24.33	FRA12C	BrdU, R	12q24.2	[86]
-	-	FRA12D	Fol, R	12q24.13	
*SELPLG*	12q24	FRA12E	Aph, C	12q24	[87]
*BRCA2*	13q12.3	FRA13A	Aph, C	13q13.2	[35]
*RB1*	13q14.2	-	-	-	[88]
*PCDH20*	13q21.2	FRA13B	BrdU, C	13q21	[89]
*PCDH20*	13q21.2	FRA13C	Aph, C	13q21.2	[89]
*ZIC2*	13q32	FRA13D	Aph, C	13q32	[90]
-	-	FRA15A	Aph, C	15q22	
*ABCC6*	16p13.1	FRA16A	Fol, R	16p13.11	[64]
*CDH1*	16q22.1	FRA16B,	Dmy, R	16q22.1	[91]
*CDH1*	16q22.1	FRA16C	Aph, C	16q22.1	[91]
*CDH13*	16q23.3	FRA16D	Aph, C	16q23.2	[92]
*WWOX*	16q23.3-q24.1	FRA16D	Aph, C	16q23.2	[93]
*HIC1*	17p13.3	-	-	-	[60]
*-*	-	FRA17A	Dmy, R	17p12	
*BRCA1*	17q21.31	-	-	-	[94]
*SOX9*	17q23	FRA17B	Aph, C	17q23.1	[95]
*CDH2*	18q12.1	FRA18A	Aph, C	18q12.2	[72]
*SERPINB5*	18q21.33	FRA18B	Aph, C	18q21.3	[96]
*BCL2*	18q21.3	FRA18B	Aph, C	18q21.3	[64]
-	-	FRA18C	Aph, C	18q22.2	
-	-	FRA19A	5azaC-R, C	19q13	
-	-	FRA19B	Fol, R	19p13	
-	-	FRA20A	Fol, R	20p11.23	
-	-	FRA20B	Aph, C	20p12.2	
-	-	FRA20B	Aph, C	20p12.2	
*FBLN1*	22q13	FRA22A	Fol, R	22q13	[97]
-	22q12.1	FRA22B	Aph, C	22q12.2	
*AR*	Xq12	-	-	-	[98]
*FMR1*	Xq27.3	FRAXA	Fol, R	Xq27.3	[14]
*VCX*	Xp22	FRAXB	Aph, C	Xp22.31	[99]
*VCX*	Xp22	FRAXC	Aph, C	Xq22.1	[99]
*FMR1*	Xq27.3	FRAXD	Aph, C	Xq27.2	[100]
*FMR2*	Xq28	FRAXE,	Fol, R	Xq28	[101]
*FMR2*	Xq28	FRAXF	Fol, R	Xq28	[101]

Definitions for standard gene abbreviations are available at the HUGO Gene Nomenclature Committee (HGNC) website [102]. Aph, aphidicolin; 5azaC-R, 5-azacytidine ; BrdU, bromodeoxyuridine; C, common; Dmy, distamycin; Fol, folate; R, rare.

## Presentation of the hypothesis

The key components of the hypothesis, presented in Figure 1, are: 1) carcinogenesis-linked hypermethylation that occurs primarily at or near fragile sites as a result of the tendency of DNA sequences at these sites to form non-B structures; 2) methylation is applied *de novo* to these structures and their neighboring sequences not only by DNMT3A/3B but also by DNMT1; 3) during normal replication methylated non-B DNA structures are returned to the B form by ERCC2, ATRX, HELLS and RecQ helicases; 4) sequences that cannot be resolved by helicase action are removed by excision; 5) hydroxymethylation applied to the nascent methyl groups by the action of TET dioxygenases prevents sequences that are resolved by helicase action from undergoing maintenance methylation by DNMT1, regenerating the unmethylated state at these sites in normal cells (in this regard, it is important to recognize that resolution of these structures will result in hemimethylated DNA, and that hemimethylated DNA is the preferred substrate of TET1 dioxygenase [17]); and 6) in addition to DNA damage, carcinogenesis-linked dysfunction among the helicases results in hypermethylation at and near fragile sites, and hypomethylation elsewhere.

## Testing the hypothesis

While the existing evidence for the proposed cycle is compelling, currently available experimental approaches permit several additional tests of the hypothesis. For example, transient knockdown by transfection-mediated expression of an ERCC2, ATRX, HELLS or RecQ helicase is predicted to result in a transient hypermethylation, coupled with an increase in local hydroxymethylation content at affected fragile sites. Stable knockdown is expected to result in both hypermethylation at affected fragile sites and global hypomethylation. In particular, the knockdown of the WRN helicase (REQL2) is predicted to result in hypermethylation of the *FHIT* gene at FRA3B [108], coupled with enhanced bisulfite sensitivity [109] of native DNA associated with the increased presence of non-B DNA structure at this site [109,110]. Existing studies on the effect of WRN mutations on methylation, for example, do not address early events at fragile sites, since they use cell lines that have been carried in culture or were isolated from adults bearing the WRN mutation [111,112]. Chromatin immunoprecipitation with antibodies to DNMT1 is expected to yield DNA that is enriched for fragile site sequences after helicase knockdown. Determining the levels of DNMT1 by immunoblotting after helicase knockdown would determine whether or not the removal of stalled DNMT1 involves proteolysis [113]. WRN knockdown coupled with DNMT1 knockdown is expected to produce enhanced bisulfite sensitivity [109,110] in the absence of hypermethylation, while enhanced bisulfite sensitivity after knockdown of DNMT1 alone would provide evidence for an obligatory role of methylation in non-B structure resolution.

Finally, as a test of the downstream portion of the cycle, overexpression of TET dioxygenases is expected to reduce *de novo* methylation at fragile sites caused by helicase knockdown, and knockdown of the dioxygenases should enhance *de novo* methylation at these same sites.

## Implications of the hypothesis

The hypothesis is consistent with other suggestions for the genesis of hypermethylation [114,115]. Disruptions in the histone code might be expected to elicit fragile site formation, since exposure to carcinogens that damage DNA or block the histone modification processes, may also induce fragile sites. Alterations in DNA structure induced by miRNA (possibly via R-loop formation) could have similar effects at these sites. Moreover, the remarkable correspondence between sites of reported hypermethylation and fragile sites suggests that the mutational and epimutational base upon which natural selection can act during carcinogenesis is largely confined to these sites. Their tendency to adopt non-B DNA structures provides a compelling case for how they become available for natural selection.

Each of the tenets of the hypothesis is supported by cytogenetic, DNA methylation and enzymological evidence. Enzymological and biological evidence from our laboratory suggests that DNMTs have evolved to recognize non-B DNA structures, like those associated with FRAXA in fragile X-linked mental retardation, and FRA11I/FRA11C in breast and prostate cancer [20,107,110], suggesting that DNMTs play an obligate role in the suppression of non-B DNA structures [116,117] along with associated repair systems. Given an obligate role for DNMTs in the suppression of non-B structure formation, the role of helicases in the process can be better understood. In general, deficiencies in helicases, such as ATRX, HELLS, BLM and WRN, have been shown to result in either global genomic demethylation [118,119], gene activation [10], or both global demethylation and gene activation. Two diametrically opposed interpretations of normal function of these helicases have been proposed. In one interpretation, they are viewed as actively promoting DNA methylation [118,120]. In the alternative interpretation, they are viewed as passively promoting normal DNA methylation by preventing the sequestration of DNMTs [107,117] at unresolved non-B structures [10]. The enzymological evidence supports the alternative interpretation. For example, the WRN helicase has been shown to resolve quadruplex DNA [15] and deficiency appears to result in the accumulation of non-B structures [10]. The evidence suggests that the DNMTs remain bound to non-B DNA sequences containing mispaired cytosines [107], oxidized bases [121] or DNA containing base analogs, such as deoxyuridine (dU) [122], 5azaC-dR or GuaUre-dR [1]. It follows, that in cases of helicase deficiency, DNMT sequestration at a site of hypermethylation will result in global hypomethylation, much like the effects of 5-azacytidine (5azaC-R**)**, 5azaC-dR and GuaUre-dR result in hypomethylation, since tightly bound DNMTs are unable to maintain normal methylation patterns. Moreover, this model (Figure 1) and the postulated obligatory role for DNMTs suggests that the cytogenetic overlap between 5azaC-R, 5azaC-dR and GuaUre-dR-induced fragile sites FRA1J and FRA9F, and the undercondensations observed in DNMT3B mutants [123] and knockouts [124], is the result of the complete titration of DNMT3B by non-B structures that remain unresolved and unrepaired after exposure to these compounds. The selective effect on DNMT3B as opposed to DNMT1 can be attributed to its low level of expression relative to DNMT1. Estimates from purification data [107] suggest that DNMT1 levels are in the order of several thousand copies per cell. Northern blotting suggests that the abundance of DNMT3B is ten to twentyfold below that of DNMT1 at a few hundred copies per cell [125]. The DNA footprint of DNMT1 is approximately 23 bp [126]. Thus, a single non-B structure involving even 1,000 bases of single-stranded DNA could sequester approximately 2% of the DNMT1 or 20% of the DNMT3B. Replication stress-inducing agents, such as aphidicolin or distamycin, can be expected to induce multiple non-B regions. During carcinogenesis, multiple rounds of sequential induction of fragile sites by replication stress and carcinogen action could result in global hypomethylation. Moreover, the shattering of metaphase that occurs at high concentrations of 5-aza-CR contrasted with the confined induction of fragile sites at low concentration [127] is consistent with the idea that DNMT3B is knocked out at low concentration and DNMT1 at higher concentration, and that both are obligatorily involved in suppressing fragile sites. Finally, work with the TET dioxygenases [16-19] and the response of DNMTs to 5-hydroxymethylcytosine (hmC) strongly suggest that hydroxymethylation at repaired and methylated genes in fragile sites will act to restore the unmethylated active state of these genes (Figure 1B).

## Abbreviations

5azaC-dR: 5-aza-2*′*-deoxycytidine; 5azaC-R: 5-azacytidine; bp: base pair; dU: deoxyuridine; DNMT: DNA methyltransferase; DNMT1: DNA methyltransferase 1; DNMT3: DNA methyltransferase 3; GuaUre-dR: 2*′*-deoxyriboguanylurea; hDNMT1: human DNA methyltransferase 1; HGNC: HUGO Gene Nomenclature Committee; hmC: 5-hydroxymethylcytosine; miRNA: microRNA; NMR: Nuclear magnetic resonance; ODN: Oligodeoxynucleotide; TET: Ten-eleven translocation.

## Competing interests

The author declared that he has no competing interests.
